# Genomic Instability in Fungal Plant Pathogens

**DOI:** 10.3390/genes11040421

**Published:** 2020-04-14

**Authors:** Shay Covo

**Affiliations:** Department of Plant Pathology and Microbiology, Robert H. Smith Faculty of Agriculture, Food and Environment, Hebrew University, Rehovot 76100001, Israel; shay.covo@mail.huji.ac.il

**Keywords:** fungal plant pathogens, genome plasticity, DNA repair

## Abstract

Fungi and fungal-like organisms (oomycetes) that cause diseases in plants have impacted human communities for centuries and probably from the dawn of agriculture. In modern agriculture, there is a constant race between new strategies to manage fungal plant pathogens and their ability to adapt. An important component in this race is fungal genetic diversity. Mechanisms such as sexual and parasexual recombination that contribute to the creation of novel allele combinations in fungal plant pathogens are briefly discussed in the first part of this review. Advances in genomics have enabled the investigation of chromosomal aberrations of agriculturally important fungal isolates at the nucleotide level. Some of these cases are summarized in the second part of this review; it is claimed that the effect of chromosomal aberrations on pathogenicity should be studied mechanistically. More data on the effect of gene copy number variations on phenotypes that are relevant to agriculture are especially needed. Genome rearrangements through translocations have shaped the genome of fungal plant pathogens by creating lineage-specific chromosome territories encoding for genes participating in plant diseases. Pathogenicity chromosomes are unique cases of such lineage-specific genetic elements, interestingly these chromosomes can be transferred horizontally and thus transforming a non-pathogenic strain to a pathogenic one. The third part of this review describes our attempts to reveal mutators in fungal plant pathogens by identifying fungi that lack important DNA repair genes or respond to DNA damage in an unconventional way. We found that a group of fungal plant pathogens lack conserved genes that are needed for an important Holliday junction resolution pathway. In addition, in *Fusarium oxysporum*, the rate-limiting step in dNTP production is not induced under DNA replication stress. This is very different from organisms from bacteria to humans. It remains to be seen if these mechanisms promote genetic instability in fungal plant pathogens.

## 1. Introduction to Fungal Plant Pathogens

### 1.1. Fungi—A Threat to Agriculture and the Environment

Fungal plant pathogens have threatened food security and imposed an economic burden since the dawn of agriculture [1]. Evidence for fungal borne diseases, such as powdery mildew and fruit rots, are found even in the Bible, and different diseases were documented by the ancient Greeks [2,3]. In modern agriculture, research-intensive breeding products are constantly being developed to increase productivity and profitability. The numerous resources invested in these products, together with monoculture practice, have reduced the genetic diversity of crops in the field. This increases the chances of a massive spread of adapted plant pathogens and the ensuing devastating damage. An example to this notion that is further discussed below is found in banana cultivation. The Gros Michel was the dominant cultivar in the first half of the 20th century and was replaced due to disease susceptibility by the Cavendish cultivar, which has now been found to be susceptible to the same disease [4]. Fungal plant pathogens severely affect food production; the biomass of staple crops that were lost in 2009–2010 due to fungal diseases could feed 8.5% of the total world population (almost twice the population of the United States) [5]. Fungal and fungal-like plant pathogens (oomycetes) have had, and are still having, devastating regional effects that can bring communities to severe economic crises, especially in developing countries. A recent fitting example is the outbreak of wheat blast caused by the fungus *Magnaporthe oryzae* in Bangladesh during 2015–2016 [6]. Fungal and fungal-like pathogens that infect trees, like *Cryphonectria parasitica* and *Phytophthora ramorum*, affect the environment directly. Since the first half of the 20th century, diseases from such pathogens have caused the loss of 100 million elm trees and 3.5 billion chestnut trees. It has been estimated that the damage fungi and oomycetes caused to trees in selected regions within less than a century would result in a cumulative loss of 230–580 megatonnes of dissolved CO_2_ [5].

The use of fungicides is probably the most important strategy to manage fungal diseases. With an estimated global market value of USD 18.7 billion in 2019, fungicides have a significant effect on our food prices [7]. Fungicides also affect human health [8] as well as biodiversity, reflected in the toxicity reports on fish and bees [9]. Moreover, the vast use of fungicides has led to an increase in resistant fungal isolates, with full or partial resistance of fungal pathogens to several pesticides [10]. Fungicides that are used to manage plant diseases are very similar to ones used in the clinic to treat patients with fungal infections. The recent report on the ubiquitous human pathogen *Aspergillus fumigatus* indicates it has developed azole-resistant isolates due to long term continuous exposure to azole-based pesticides, leading to failure of azole treatment in infected humans [11]. This increases the concern regarding the indirect effect of fungicides on human health.

Breeding for crops resistant to fungal-borne diseases could be an alternative to fungicides. However, it is a time consuming and expensive process which has only provided solutions for some pathogens. Moreover, like pesticide resistance, there is a risk of pathogen adaptation to the resistant crop. This risk is best exemplified in two cases that have alerted the entire agricultural research community and, more importantly, farmers and consumers worldwide. Panama disease, caused by the ascomycete fungus *F. oxysporum* f. sp. *cubense,* almost destroyed banana cultivation in Central America in the mid-1900s. Eventually, the major banana cultivar Gros Michel was replaced by Cavendish, a variety that was resistant to *F. oxysporum* f. sp. *cubense* Tropical Race 1 [4]. However, in the last decade, another isolate, *F. oxysporum* f. sp. *cubense* Tropical Race 4, emerged with the ability to cause disease in Cavendish and is now spreading throughout banana-cultivation areas [12,13]. The other example is the Ug99 isolate of the basidiomycete wheat pathogen *Puccinia graminis*, which became adapted to resistant wheat and is now causing significant losses in Africa and the Middle East [14]. Adaptation of pathogens to crops and pesticides is often due to the generation of novel allele combinations. In the next paragraphs, mechanisms that increase genetic diversity by forming new allele combinations are briefly described.

### 1.2. Genetic Recombination in Fungal Plant Pathogens

While at a given time many pathogens spread clonally, from an evolutionary standpoint, genetic exchange is favorable in fungal plant pathogens [15]. Fungi create new combinations of alleles by genetic exchange. Previous reviews discuss extensively how genetic exchange between fungal isolates, whether via sexual recombination, parasexual recombination, or hybridization, contributes to pathogenesis (reviewed in [5,16,17]). Here we will only provide a few examples and highlight some of the emerging questions.

There is evidence for the importance of sexual recombination to plant pathology. For example, sexual recombination was shown to be important for pesticide resistance of the Oculimacula species. Since the 1970s, cereals were treated in the UK with benzimidazoles to prevent Eyespot disease caused by Oculimacula species. Infrequent resistant isolates were found in treated fields with limited distribution. During the early 1980s, the frequency of resistant isolates and their distribution increased [18]. The change was attributed to the appearance of telemorph (sexual stage) isolates [18]. One explanation to this phenomenon is that the resistant isolates that propagated clonally only had an advantage when the pesticide was locally applied. When the use of the pesticide stopped, the resistant isolates were outcompeted. Introducing sexual recombination enabled the creation of new isolates with an allele combination that was on the one hand pesticide-resistant, but on the other hand, could spread better in the fields. In addition, the wind-borne ascospores (spores that are the product of meiosis in ascomycetes) dispersed much further than the asexual conidia that were dispersed only by raindrops [18]. One of the best examples for the role of sexual recombination in plant pathology is found in the wheat pathogen *Zymoseptoria tritici* [16,19,20]. The pathogen exhibits high genetic diversity with hallmarks of sexual reproduction [16,20,21] (and the references therein). For example, a study had shown that the percentage of recombinants between two genotypes increased during the growing season. The recombinant isolates were even more successful at infecting moderately resistant crops than the parental ones [19]. Quantitative trait loci analyses revealed that the sexual life cycle driven genetic diversity allowed the rapid adaptation of the pathogen to region-specific management practices and cultivars (reviewed in [16]).

For many fungal plant pathogens, a sexual life cycle has never been observed or it has been only rarely observed. Nevertheless, in some of these species, parasexual recombination was demonstrated [22,23]. Parasexual recombination is defined as a genetic exchange between cells without meiosis. In general, parasexual recombination occurs in filamentous fungi through the fusion of hypha (anastomosis) followed by a fusion of nuclei. The diploid-like nuclei that are formed are unstable; eventually, chromosomes are lost to form a haploid nucleus that contains chromosomes from the two parental nuclei and potentially even recombined chromosomes [24,25]. Parasexual recombination performed in vitro in *F. oxysporum* lead to the creation of a new combination of alleles in the daughter cells that are important for pathology [26]. Nevertheless, the role of parasexual recombination in the epidemiology of plant diseases is not clear yet. To the best of my knowledge, parasexually-driven allele combination that conferred virulence, adaptation to the host, or pesticide resistance in the field has not yet been reported. One interesting phenomenon that falls within the category of parasexual recombination is the transfer of chromosomes between *F. oxysporum* isolates as will be described in detail below.

Hybrids, which are defined as offspring of two non-conspecific individuals, have a role in the evolution and emergence of new fungal plant pathogens [17]. An example of a unique hybridization scenario that does not fit the classic definition above was reported for the rust fungus *P. graminis* Ug99. Rust fungi are very important pathogens for cereals. These species have a complex life cycle that includes both sexual and asexual stages, the latter contributing to genetic diversity [27]. However, many rusts can reproduce clonally through in planta propagation of dikaryotic urediniospores (the most common asexual spores of rust species) [28]. A recent study showed that the Ug99 isolate of *P. graminis* emerged through somatic hybridization of nuclei within the dikaryon arrangement [14,28]. By sequencing the isolates of Ug99 and *P. graminis* race 21 (Pgt21), the authors determined that both isolates share almost the same haplotype for one of the nuclei. The other nucleus was very different between the isolates. The authors demonstrated that the genome of Ug99 was the result of somatic hybridization between the Pgt1-21 nucleus and another as yet unidentified nucleus [28]. This scenario was more probable than sexual recombination based on the sequence reads and the physical maps of the chromosomes created by Hi-C sequencing [28].

Chromosomal aberrations and mutations that are generated de novo during mitosis [29] or meiosis [30] cause many important phenotypes in human and other organisms. The same is potentially true for fungicide resistance and hyper-virulence in fungal plant pathogens. The rest of this review will focus on chromosomal aberrations that lead to genetic diversity and fungal adaptability.

## 2. Chromosome Plasticity—Implications in Plant Pathology

### 2.1. Aneuploidy and Copy-Number Variations (CNVs)

One of the most common aberrations in fungal pathogens of humans is aneuploidy [31,32,33]. Aneuploidy is the presence of an uneven number of chromosomes resulting from chromosome loss or gain. There are not many examples of aneuploidy in fungal plant pathogens. This might be because the human pathogen *Candida albicans* is a diploid; *Cryptococcus neoformans* is haploid but in some cases, pathogenicity related phenotypes are associated with aneuploidy derived from polyploid genome [33,34]. In contrast, most fungal plant pathogens are haploids, and therefore core chromosome loss is lethal, and chromosome gain has a relatively high effect on gene dosage [35]. Oomycetes are fungal-like organisms that cause important plant diseases and possess a diploid karyotype [36]. In these organisms, aneuploidy has been previously reported [37,38]. Especially interesting are the reports concerning the oomycete *P. ramorum*, the causal agent of Sudden Oak Death [37,39]. In those reports, it was shown that the phenotype and karyotype of strains isolated from diseased trees were different than the ones grown in vitro. Oomycetes isolated from Oak trees, in this case, showed partial aneuploidy. Therefore, the authors described the phenomenon as host-induced aneuploidy [37]. However, the mechanisms of this host-induced aneuploidy remain unclear. It is also unclear whether the reason for the aneuploidy is a decrease in chromatid-transmission fidelity, strong selection for adapted aneuploid isolates, or both. Aneuploidy was also observed in several isolates of the fungal pine pathogen *Dothistroma septosporum*. Eighteen isolates from different sites around the world were re-sequenced; many changes in the chromosomes were found, including deletions, translocations, and partial and complete aneuploidy [40]. Interestingly, the authors correlated aneuploidies with strains that overproduced dothistromin [40], a mycotoxin that is important for pine pathology but also has a genotoxic effect on humans [41,42,43]. It is expected that more re-sequencing projects of fungal plant pathogens will identify more cases of aneuploidy. The challenges are to understand how aneuploidy is generated, the role of the interaction with the host, and whether aneuploidy is beneficial and, if so, why.

Aneuploidy is an extreme case of gene CNV. Other examples of CNV have been reported in several fungal plant pathogens, including some good examples for a direct connection between CNV and a phytopathogenic capability. Powdery mildew caused by *Erysiphe necator* is a devastating disease in grapes. The disease is managed by the application of sterol demethylase inhibitors (DMIs) and other fungicides. A known mode of resistance to DMIs is a point mutation in the target gene *EnCYP51*; however, re-sequencing DMI-resistant *E. necator* isolates revealed frequent increases in copy number of the mutant allele [44]. The authors showed that the increase in *EnCYP51* copy number was correlated with increased gene expression. In addition, isolates with high copy numbers of the mutated *EnCYP51* gene were much more prevalent in DMI-treated vineyards than in untreated ones [44]. Interestingly, while many examples have been reported for base substitutions in genes that confer resistance to fungicides, there are very few examples of increased copy number. Overexpression of drug-efflux pumps was observed in fungi resistant to fungicides, but to the best of our knowledge, an increased copy number of the respective efflux pump genes has not been reported in fungal plant pathogens [45,46].

### 2.2. Structural Variations

Structural variations include deletions, inversions, and translocations that occur in the genome relative to a reference genome. An increased number of cases of structural variations in fungal plant pathogens have been recently reported [40,47,48,49]. A groundbreaking paper compared the genomes of several pathogenic and non-pathogenic isolates of *Verticillium dahliae* and found very little difference in their gene sequences [47]. In contrast, the authors demonstrated extensive structural variations between the isolates, mainly translocations. The chromosomal rearrangements created lineage-specific segments that were associated with pathogenicity [47]. The breakpoints of the structural variations were enriched with repetitive elements that had been previously demonstrated to facilitate structural variations in *Saccharomyces cerevisiae* [50,51].

Taphrina is a genus from the subphylum of Taphrynomycotina. The genus includes several plant pathogenic species, amongst them the leaf curl disease-causing agent *Taphrina deformans*. Recently, a comparative genome analysis of Taphrina species has been conducted [49]. The analysis revealed that pathogens of the same host family were phylogenetically clustered. Synteny analysis has revealed extensive chromosomal rearrangements between the different species [49]. The rearranged genome loci constitute lineage-specific domains that are enriched in repetitive elements [49], supporting the notion that inaccurate double-strand breaks due to repeats shape the evolution of lineage-specific regions. Such inaccurate repair could be due to homologous recombination between ectopic repetitive sequences rather than the sister chromatid [50] or due to the fusion of ectopic repetitive sequences by a mechanism known as single-strand annealing [52]. The lineage-specific regions were enriched for genes that were upregulated during plant infection, associating these genes with plant disease. Further analysis had revealed that the genomic regions that showed high plasticity between Taphrina species were enriched in genes that were predicted to encode for effectors (proteins that manipulate host cells during infection) [49].

Lineage-specific regions enriched with repetitive sequences have been described in plant pathogens from different subphyla—Taphrina species from Taphrinomycotina and Verticillium and Fusarium species from Pezizomycotina [47,49,53]. Therefore, it is likely that genomic regions with a high density of repetitive elements are hot spots for the creation of pathogenicity associated lineage-specific genomic regions. The underlying mechanisms that enabled the formation of these regions, which are seeds for genomic innovation, are still unclear.

### 2.3. Dynamics of Accessory Chromosomes in Fungal Plant Pathogens

Probably the most interesting aspect of chromosome plasticity in fungal plant pathogens is the biology of the accessory chromosomes. Several fungal and fungal-like plant pathogens contain chromosomes that can be lost and are therefore referred to as accessory, dispensable, or supernumerary [53,54,55]. In some cases, the accessory chromosomes encode genes that are essential for pathogenicity and are therefore called pathogenicity chromosomes [53,55,56,57]. In these cases, the genome is divided between core chromosomes that encode most of the biochemical activities in the cell and the pathogenicity chromosomes.

The accessory/pathogenicity chromosomes of *F. oxysporum* f. sp. *lycopersici* (*Fol*) were studied in detail at the genomic level. Using comparative genomics, Ma et al., revealed that *Fol* contains four chromosomes with no synteny to phylogenetically related Fusarium species [53]. While these chromosomes are enriched with transposable elements and are probably heterochromatic, one of them, chromosome 14, encodes Secreted In Xylem (SIX) genes that are important for pathogenicity [58]; complete loss of chromosome 14 resulted in the loss of pathogenicity [59]. The rate of loss of chromosome 14 under axenic growth conditions was estimated at 1:65,000 [59]. This is far lower than the rate of loss of *Z. tritici* dispensable chromosomes, measured at 1–3% for some of them [48]. A more detailed investigation of the consequences of chromosome 14 plasticity in *Fol* revealed that pathogenicity is maintained even when 900 Kb of the chromosome are lost, including some of the SIX genes [59]. A sexual life cycle has never been reported for *Fol*; therefore, it was interesting to determine whether the pathogenicity chromosomes can be exchanged in a parasexual manner between different strains. Strikingly, Ma et al., [53] showed that chromosome 14 of *Fol* can be transferred; this transfer was enough to turn a non-pathogenic isolate into a pathogenic one. As described above, this probably occurs through anastomosis and nuclear-fusion (parasexual recombination). Recently, it has been shown that the ability of chromosome 14 to be transferred is rather unique; other chromosomes, and even accessory chromosomes, either cannot be transferred or are transferred at a frequency that is far lower than that of chromosome 14 [60]. Interestingly, at a very low frequency, core chromosomes, or parts of them, can also be transferred, but always together with chromosome 14. When a core chromosome, or part of it, is transferred from a donor fungus, there is a corresponding loss of genetic material in the recipient fungus, thereby preventing aneuploidy [60]. This evidence suggests that homolog pairing occurs within the fused nucleus. It is unknown what, if at all, is the role of this pairing in the process. The ability to transfer pathogenicity chromosomes is not restricted to *Fol*; it was also observed in the closely related cucurbit pathogen species *F. oxysporum radicis-cucumerinum* and probably in *Alternaria alternata* [56,61]. Chromosome transfer without transfer of pathogenicity has been observed in other species [62]. Understanding the molecular mechanism of chromosome transfer in fungal plant pathogens still remains a challenge for cell biologists, geneticists, and evolutionary biologists interested in plant diseases.

## 3. Comparative Genomics Generate Hypotheses Regarding Causes of Genome Plasticity in Fungal Plant Pathogens

### 3.1. Genomic Analyses of the DNA Damage Response in Fungal Plant Pathogens

In the last two decades, there have been at least two excellent reviews regarding possible mechanisms of genome instability in fungal plant pathogens [63,64]. In these and other reviews, the initiating event leading to genome instability was thought to be damage to DNA (usually in the form of strand breaks). DNA damage from external and internal sources induces genome instability in many organisms [50,52,65,66]. Accurate DNA repair protects cells from DNA damage-induced genome instability [67]. In agreement, mutants in DNA repair genes exhibit a high rate of genomic instability [68,69,70]. Isolates of pathogenic bacteria are often mutators, where they exhibit high mutation rates and are mutated in DNA repair genes [71,72]. To the best of my knowledge, there is no documentation of fungal plant pathogen field isolates that are mutated in DNA repair genes. We tried to identify putative mutators in fungal plant pathogens by examining the DNA repair gene repertoire of dozens of fungal plant pathogens and by investigating the response to DNA damage of *F. oxysporum*. The main findings are summarized below.

**Figure 1:**
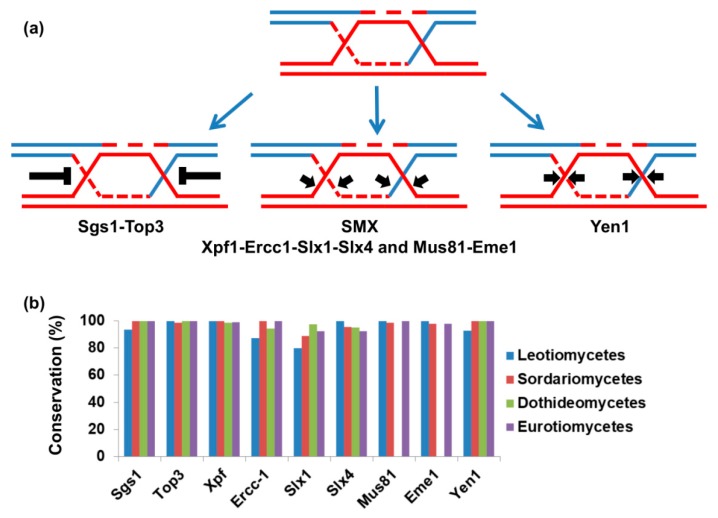
Mechanisms of Holliday junction resolution (**a**). Four evolutionarily conserved mechanisms to resolve Holliday junctions or similar joint molecules. Holliday junction dissolution: RecQ helicase (Sgs1) facilitates the migration of two Holliday junctions toward each other to yield a hemicatenane structure that is relieved by topoisomerase III. The second and third mechanisms of Holliday junction resolution are based on the activity of the SMX complex: a complex of three nucleases participates in introducing nicks into the Holliday junction, thus resolving them. The complex is composed of Xpf-Ercc1, Slx4-Slx1, and Mus81-Eme1 but Mus81-Eme1 also acts independently. The fourth mechanism of Holliday junction resolution is based on the activity of Yen1 that introduces asymmetric incisions across the joint junctions; (**b**) using comparative genomics; we identified the repertoire of Holliday junction resolution genes in species from Pezizomycotina (classes Leotiomycetes, Sordariomycetes, Dothideomycetes and Eurotiomycetes). Conservation represents the fraction of species that encode the genes shown on the *X*-axis in each class.

Recently, it was reported that the genus of Hanseniaspora, from the phylum of ascomycetes, exhibited a very limited DNA repair gene repertoire and correspondingly high evolution rate [73]. We performed a comparative genomic investigation of DNA repair genes in over 300 species of fungi from ascomycetes including dozens of plant pathogens [74]. We did not observe a genus of fungal plant pathogens that was equivalent to Hanseniaspora from evolution rate and DNA repair gene repertoire standpoints [74]. Nevertheless, further careful analyses and many more species are needed to determine if there is a genus of fungal plant pathogens that has a mutator genotype. We observed a group of organisms that lost important DNA repair genes. All of the tested species of the class of Dothideomycetes, including *Z. tritici*, *A. alternata*, *Venturia inequalis*, *Cochliobolus heterostrophus*, and *D. septosporum*, lack enzymes that resolve homologous recombination intermediates (Figure 1) [74]. (More about homologous recombination and fungal genome stability can be found in this issue [75]).

### 3.2. Resolution of Holliday Junctions

Homologous recombination is needed to repair double-strand breaks in the genome, whether they are generated by DNA damage or programmed as part of meiosis initiation [76,77]. One important role of homologous recombination is the restart of replication forks that have stalled due to lesions in the DNA, replication-borne breaks, or secondary DNA structures [78,79,80]. If not properly resolved, intermediates of homologous recombination put genome stability at risk [79,81,82,83]. During homologous recombination, joint molecules are generated between the invading DNA molecule and the template for recombination (these structures are also known as Holliday junctions) [76]. Joint molecule structures that may differ from Holliday junctions are formed during replication restart [84]. Holliday junctions have to be resolved before anaphase; otherwise, chromatid nondisjunction or breakage will occur [84,85,86]. There are four evolutionarily conserved pathways that deal with Holliday junctions or similar joint molecules (illustrated in Figure 1a and reviewed in [87]). The first is Holliday junction dissolution by helicase from the RecQ superfamily and DNA topoisomerase III. In this case, the two DNA molecules are separated without the need for nuclease activity [88]. The other three mechanisms resolve Holliday junctions using endonuclease activity; these enzymes are known as Holliday junction resolvases or structure-specific endonucleases [87]. Two of these mechanisms cooperate in the same complex (the SMX complex) [89]. The SMX complex is composed of the nucleases XPF-ERCC1 and SLX4-SLX1 and MUS81-EME1 nucleases although MUS81-EME1 can operate independently [87,89]. Mus81 activation is needed to resolve joint molecules created during DNA replication; without this activity, cells may contain anaphase bridges—structures that have been shown to induce genome instability [90]. The fourth mechanism to resolve Holliday junctions is Yen1, which is also a resolvase that functions separately from the SMX complex [91]. There is partial redundancy between the different mechanisms that resolve homologous recombination intermediates [91,92,93]. However, in *S. cerevisiae*, *mus81*-null mutants are much more sensitive to DNA damage than yen1-null mutants [94].

Despite the conservation of MUS81-EME1 and the importance of the complex in eukaryotes, the entire class of Dothideomycetes lacks both proteins (Figure 1b) [74]. Other mechanisms to resolve recombination intermediates are conserved (Figure 1b) [74]. The functional significance of this is not yet known. It is possible that either Dothideomycetes pay the price for the lack of MUS81-EME1 in genome instability, or that in these organisms, alternative mechanisms have been adapted. The high rate of loss of accessory chromosomes in *Z. tritici* and the high rate of aneuploidy described in *D. septosporum* may indicate inaccurate chromosome-transmission machinery, as expected from the absence of MUS81-EME1 [40,48]. The next section will focus on the response of *F. oxysporum* to DNA damaging agents that cause DNA replication stalling and breaks, therefore, provoking genome instability.

### 3.3. Deoxyribonucleotide Triphosphate (dNTP) Biosynthesis in Response to DNA Replication Stress

**Figure 2:**
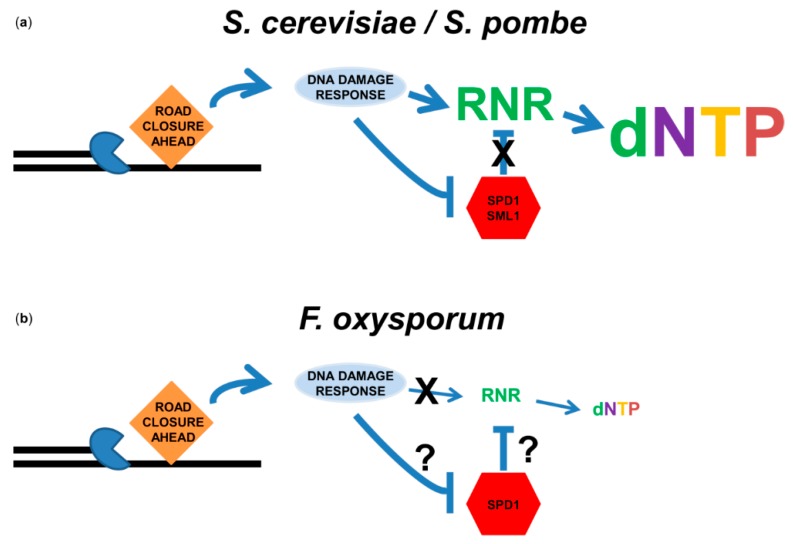
DNA-replication stress does not induce dNTP production in *F. oxysporum.* (**a**) Role of the DNA-replication-stress/DNA-damage response in dNTP pool elevation in yeast. Upon stress to the replication fork, single-strand gaps are exposed and DNA-damage signaling pathways are activated. Either canonical (*S. pombe*) or DNA-damage-specific (*S. cerevisiae*) RNRs are induced at the transcriptional level. In addition, RNR inhibitors Spd1 (*S. pombe*) or Sml1 (*S. cerevisiae*) are degraded. The result is an increase in the dNTP pools. (**b**) Role of the DNA-replication-stress/DNA-damage response in dNTP pool elevation in *F. oxysporum*. Although the DNA-damage response was activated by MMS and hydroxyurea in *F. oxysporum*, induction of RNR was not observed at either the transcriptional or translational levels. Consequently, the dNTP pool was not increased in response to DNA damage. The role of *F. oxysporum* Spd1 in dNTP pool determination remains unknown.

Our comparative genome analysis of DNA-repair genes across 300 ascomycete species revealed that *F. oxysporum* has a vast repertoire of DNA-repair genes [74]. Nevertheless, previous reports have suggested that the population of *F. oxysporum* is diverse, exhibiting multiple chromosomal aberrations [95,96]. Genome instability could arise from an inefficient DNA-damage response [97]. Nevertheless, transcriptome analysis of germinating conidia exposed to the DNA damaging agent methyl methanesulfonate (MMS) revealed a strong response of multiple DNA-repair enzymes, including genes that participated in excision repair, homologous recombination, Holliday junction resolution and non-homologus end-joining [98]. The DNA damage signal transduction pathway was also induced, starting with induction of the 911 DNA-damage-checkpoint complex and continuing with phosphorylation of DNA-damage transducers Chk2 and Chk1 [98]. However, one observation stood out: an increase in the expression of ribonucleotide reductase (RNR) in response to DNA damage was not observed [98]. RNR catalyzes the rate-limiting step in deoxyribonucleoside monophosphate (dNMP) production and is tightly regulated on several layers [99,100,101]. In organisms ranging from *Escherichia coli* to humans, RNR is induced by DNA damage [102,103,104,105,106]. In *S. cerevisiae* and *Schizosaccharomyces pombe*, RNR activity is inhibited by Sml1 and Spd1, respectively [107,108]. The DNA-damage response targets these inhibitors for degradation [109,110]. While Spd1 is conserved among filamentous fungi (Almog and Covo unpublished results) how it is regulated is unknown. The increased activity of RNR due to induction and inhibitor degradation causes, in many cases, an increase in the dNTP pools. This induction is thought to allow efficient DNA synthesis associated with DNA repair, or efficient replication following DNA damage [106,110,111]. Thus, presumably, the lack of induction of RNR can result in lower repair capacity and a higher rate of stalled replication forks. Induction of RNR in *F. oxysporum* at either the RNA or protein levels was not observed (Figure 2b) [98], not even when cells were exposed to the inhibitor of the enzyme hydroxyurea [112]. Unlike other organisms coming from different domains of life, there was no induction of the dNTP pools following exposure to DNA damage [112] (Figure 2b). There was a reduction in the pool following exposure to hydroxyurea [112]. Whether or not the results presented here represent the status in other fungi is unknown. Insufficient dNTP pools are associated with genome instability in humans and *S. cerevisiae* [113,114]. We are currently investigating whether the dNTP response to replication stress is a significant factor in the maintenance of genome stability in *F. oxysporum.*

## 4. Conclusions

Research into the genomic dynamics of fungal plant pathogens is in its infancy. There is a need to re-sequence many more agriculturally relevant isolates. Due to the repetitive nature of some fungal genomes, they need to be analyzed using long-read sequencing platforms [28,60]. A comprehensive genomic analysis of fungicide-resistant fungi may change our view of resistance mechanisms and lead to a change in fungicide development. An investigation into the mechanisms of mutation formation is also needed. In bacteria, such investigations led to the identification of two potential targets for reducing bacterial evolvability [115,116]. Might it also be possible to reduce the evolvability of fungal plant pathogens? Would such a reduction extend the effectiveness of fungicides or resistant crops at least for some pathogens?
